# Multiple Aneurysms of the Inferior Pancreaticoduodenal Artery: A Rare Complication of Acute Pancreatitis

**DOI:** 10.1155/2013/621350

**Published:** 2013-02-24

**Authors:** Chris Klonaris, Emmanouil Psathas, Athanasios Katsargyris, Stella Lioudaki, Achilleas Chatziioannou, Theodore Karatzas

**Affiliations:** ^1^Second Department of Propaedeutic Surgery, University of Athens Medical School, “Laikon” Hospital, 17 Ag. Thoma Street, 11527 Athens, Greece; ^2^Department of Radiology, University of Athens Medical School, “Areteion” University Hospital, 76 Vassilissis Sofias Str., 11528 Athens, Greece

## Abstract

Inferior pancreaticoduodenal artery (IPDA) aneurysms are uncommon, representing nearly 2% of all visceral aneurysms, and sporadically associated with celiac artery stenosis. Multiple IPDA aneurysms have been rarely reported. We report a case of a 53-year-old female patient with a history of prior pancreatitis, who presented with two IPDA aneurysms combined with median arcuate ligament-syndrome-like stenosis of the celiac trunk. The patient was treated successfully with coil embolization under local anesthesia. The procedure is described and illustrated in detail and the advantages and technical considerations of such an approach are also being discussed.

## 1. Introduction

Aneurysms of the inferior pancreaticoduodenal artery (IPDAA) represent about 2% of all visceral artery aneurysms and are typically associated with pancreatic or biliary tract disease [1]. Although rare, IPDA aneurysms tend to rupture quite often and unlike other splanchnic artery aneurysms, there is no clear correlation between the size of PDAAs and rupture, which occurs in up to 75% of cases [2]. Thus, incidental diagnosis of asymptomatic IPDAAs warrants prompt evaluation and treatment. Due to their anatomical location in surgically inaccessible regions and the often coexisting pancreatic infection, open surgical repair is challenging even in cases without rupture [3]. Endovascular techniques provide an attractive alternative treatment option with minimal morbidity for patients presenting with IPDAAs. We report on the management of a female patient that was presented to our department with two IPDA aneurysms (26 mm and 20 mm in diameter) two years after an episode of gallstone pancreatitis. 

## 2. Case Report

**Figure 1:**
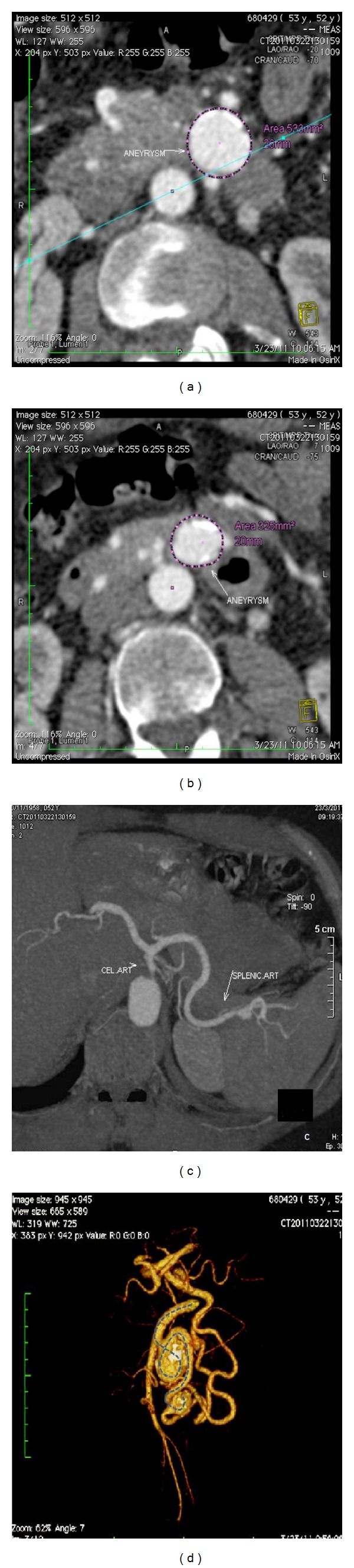
(a, b) Intrapancreatic aneurysms of the IPDA on CTA. (c) Median arcuate ligament-syndrome-like stenosis of the celiac trunk origin. (d) 3D volume rendering image processing provides closeup of the superior mesenteric artery, both IPDA aneurysms and their connecting branch.

A 53-years-old female patient was being evaluated by her physician for atypical dyspeptic symptoms over the past eight weeks. Her medical history included gallstone pancreatitis and open cholecystectomy two years previously. At presentation, she was asymptomatic with unremarkable laboratory profile. Duplex ultrasonography of the abdomen revealed two intrapancreatic formations that represented arterial aneurysms. A CTA of the abdominal aorta and splanchnic arteries revealed two saccular aneurysms of the inferior pancreaticoduodenal artery (IPDA), 26 mm and 20 mm in diameter, respectively (Figures 1(a) and 1(b)). These findings were combined with median arcuate ligament-syndrome-like stenosis of the celiac trunk (Figure 1(c)). 

Due to the location of the IPDA aneurysms, as well as patient's refusal to primarily undergoing open surgical repair, she was referred to our department for endovascular treatment. Further image processing with 3D volume rendering demonstrated the exact anatomy of the pancreatic arterial arcade and allowed for interventional planning, since both aneurysms looked morphologically suitable for coil embolization—saccular with narrow neck—and were also connected with a small collateral branch (Figure 1(d)—dashed line). Informed consent was obtained and we proceeded with coil embolization of both aneurysms.

**Figure 2:**
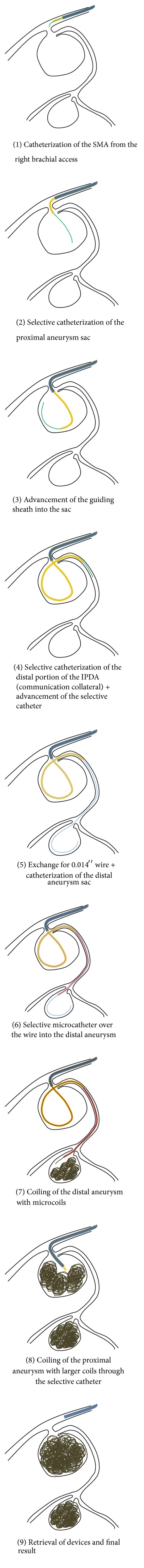
Diagram demonstrating the steps of the procedure. SMA: superior mesenteric artery, colors—blue: guiding sheath, yellow: selective catheter, green: 0.035′′ hydrophilic guidewire, red: microcatheter, Cyan: 0.014′′ guidewire, olive gray: coils.

**Figure 3:**
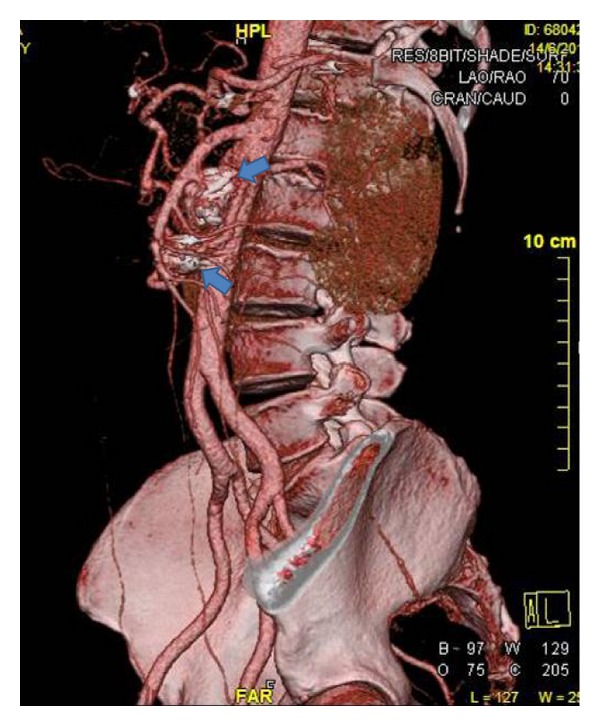
Follow-up imaging with volume-rendering lateral CTA views showing patent SMA and branches with thrombosis of both IPDA aneurysms and no sac reperfusion.

The procedure was performed in the operating room with a C-Arm (Philips, BV 300) under local anesthesia via right brachial access. After intravenous administration of 5.000 IU of heparin, a 6 Fr guiding sheath (Arrow International, Inc., PA, USA) was advanced to the level of the superior mesenteric artery (SMA). The ostium of the SMA was initially catheterized using a 0.035′′ hydrophilic stiff Terumo guidewire and a 5F long selective multipurpose catheter using standard coaxial technique (Figure 2(1)). Subsequently, the proximal aneurysm sac was catheterized and the guiding sheath was advanced into it in order to provide additional support for further maneuvers (Figure 2(2) and (3)). Thereafter, we attempted to catheterize the communicating collateral branch leading to the distal aneurysm. To do so, the wire and the selective multipurpose catheter had to follow a circular route around the sac, since the ostium of the collateral communicating branch was in close proximity and in a steep angle to the aneurysm neck (Figure 2(4)). With the multipurpose catheter placed at the upper proximal part of the communicating branch, the 0.035′′ hydrophilic guidewire was exchanged for a BMW 0.014′′ wire (Guidant Corporation, Temecula, CA), and the distal IPDA aneurysm was catheterized (Figure 2(5)). A 3F microferret microcatheter (COOK Inc., Bloomington, IN, USA) was then advanced into the distal aneurysm (Figure 2(6)), through which we proceeded to coil embolization with Hilal Embolization Microcoils (COOK Inc., Bloomington, IN, USA) (Figure 2(7)). A total of 30 microcoils were ultimately used. Thereafter, the microcatheter was withdrawn and the multipurpose selective catheter was pulled back into the proximal aneurysm sac. The latter was successfully embolized with larger 15 mm MReye Embolization Coils (COOK Inc., Bloomington, IN, USA) (Figure 2(8)). After successful coiling of both aneurysms, the whole system was retrieved and hemostasis was achieved with manual compression (Figure 2(9)). The whole procedure lasted for 94 minutes with total fluoroscopy time of 38 minutes and minimal blood loss (<50 mL). The patient was sent back to the ward and was discharged the following day. Follow-up imaging with CTA at 1 and 3 months postoperatively revealed patent SMA with preservation of collaterals and successful thrombosis of both IPDA aneurysms without any signs of sac reperfusion or enlargement (Figure 3—blue arrows). The patient remained well 18-month after intervention and is being followed up with duplex ultrasound studies on a 6-month basis.

## 3. Discussion

Aneurysms of the pancreaticoduodenal artery (PDA) are rare and most of the time present with rupture, intra-abdominal hemorrhage, or pancreaticus [4]. Mortality in ruptured PDAAs is high, approximating 29% of cases [5], making thus their early detection and prompt treatment mandatory. Asymptomatic PDA aneurysms are diagnosed incidentally during abdominal ultrasound or CT/MRI for other indications. Apart from atherosclerosis PDA aneurysms may be also due to other etiologies including pancreatitis, biliary disease, fibrodysplasia, trauma, and congenital anomalies. The coexistence of a IPDAA with celiac trunk stenosis or occlusion has been well described [6], although the presence of multiple aneurysms—as in our case—is extremely rare [7]. 

Open surgical repair includes a variety of major operations, ranging from simple ligation with or without revascularization to partial pancreaticoduodenectomy [3]. Mortality rates are high and reported to be up to 19% [8]. On the other hand, endovascular methods including cyanoacrylate glue thrombosis, aneurysm exclusion using a stent graft, and embolization with intravascular coils or detachable balloons [9] provide an attractive alternative with minimal morbidity and mortality compared to open surgery [10]. 

The use of coils in particular offers many advantages over other endovascular techniques, mostly because of the precision in their deployment and preservation of collateral branches. Nevertheless, selective catheterization of the sac and coiling can be challenging and has also anatomical limitations. In cases of saccular aneurysms, the diameter of the neck and the neck-sac diameter ratio is a crucial point and should not exceed 3 mm and 1.5, respectively, in order to avoid embolization of the coil outside the sac to distal arterial branches [11].

Careful examination of the 3D-CTA images in multiple views can help planning a safe endovascular approach and provides adequate information for the exact anatomic localization of these lesions and especially their correlation to the pancreas that is very important in case of open conversion [12]. With the advent of modern CT and MR angiography, visceral artery aneurysms less than 1 cm in diameter are routinely detected. Multislice computed tomography angiography is also a convenient imaging modality for following up after embolization, although it frequently presents with artifacts in cases of coiling and angiography is occasionally mandatory to exclude aneurysm reperfusion. Duplex ultrasonography performed by a vascular specialist is also an accurate method to follow up these patients and provides hemodynamic information for the whole visceral circulation [13].

Regarding the coexistence of celiac trunk stenosis and PDAAs, Sutton and Lawton [14] have proposed that hemodynamic changes due to celiac artery stenosis could cause aneurysm formation in the PDA. Nevertheless, during our 18-month followup, no new aneurysm or dilatation in the pancreatic arcade was noticed. Stenosis of the celiac trunk in our case was considered to be due to the compression from the median arcuate ligament rather than atherosclerotic, and therefore endovascular intervention either with PTA or stenting of the celiac trunk origin was precluded. Furthermore, due to the asymptomatic nature of the stenosis, we decided to follow up this patient rather than offering open repair.

## 4. Conclusion

Although inferior pancreaticoduodenal artery aneurysms (IPDAAs) represent a rare entity, they tend to rupture quite often and, unlike other splanchnic artery aneurysms, there is no clear correlation between size and rupture [2]; thus, their detection mandates prompt treatment. Selective embolization of these lesions is a less invasive procedure with minimal mortality and good results [9], although long-term followup is recommended. Multislice computed tomography angiography offers an accurate diagnosis and valuable information for preoperative planning [12].
